# DOA Estimation for Underwater Wideband Weak Targets Based on Coherent Signal Subspace and Compressed Sensing

**DOI:** 10.3390/s18030902

**Published:** 2018-03-18

**Authors:** Jun Li, Qiu-hua Lin, Chun-yu Kang, Kai Wang, Xiu-ting Yang

**Affiliations:** 1 School of Information and Communication Engineering, Faculty of Electronic Information and Electrical Engineering, Dalian University of Technology, Dalian 116024, China; qhlin@dlut.edu.cn; 2 Department of Underwater Weaponry and Chemical Defense, Dalian Naval Academy, Dalian 116018, China; dlkangcy@163.com (C.K.); yangxiuting2003@163.com (X.Y.); 3 Department of Computer Science and Technology, Dalian Neusoft University of Information, Dalian 116023, China; wangkai2006@neusoft.edu.cn

**Keywords:** direction of arrival estimation, underwater acoustics, compressed sensing, reconstruction algorithm, wideband

## Abstract

Direction of arrival (DOA) estimation is the basis for underwater target localization and tracking using towed line array sonar devices. A method of DOA estimation for underwater wideband weak targets based on coherent signal subspace (CSS) processing and compressed sensing (CS) theory is proposed. Under the CSS processing framework, wideband frequency focusing is accompanied by a two-sided correlation transformation, allowing the DOA of underwater wideband targets to be estimated based on the spatial sparsity of the targets and the compressed sensing reconstruction algorithm. Through analysis and processing of simulation data and marine trial data, it is shown that this method can accomplish the DOA estimation of underwater wideband weak targets. Results also show that this method can considerably improve the spatial spectrum of weak target signals, enhancing the ability to detect them. It can solve the problems of low directional resolution and unreliable weak-target detection in traditional beamforming technology. Compared with the conventional minimum variance distortionless response beamformers (MVDR), this method has many advantages, such as higher directional resolution, wider detection range, fewer required snapshots and more accurate detection for weak targets.

## 1. Introduction

Passive estimation of target direction in sonar imaging, also called direction of arrival (DOA) estimation, does not need to transmit signals and thus ensures good concealment for the delivery platform. Such estimation methods use the noise signal of target radiation to determine the target orientation. Substantial attention has been directed to DOA estimation of underwater targets, and long-term efforts have been made in both theory and experiment.

Typical DOA estimation methods include conventional beamformers [1,2], minimum variance distortionless response beamformers (MVDR) [3,4,5,6,7,8,9,10,11,12,13,14,15,16], and multiple signal characterization beamformers (MUSIC) [17,18]. The conventional beamformer based on the delay-and-sum approach has low directional resolution. In 1969, Capon [3] proposed an adaptive beamformers which is the prototype of the MVDR method. The Capon beamformers [3,4,5,6] have better resolution and interference rejection capability than the conventional beamformers, but the performance of the Capon beamformer is also sensitive to the estimation error, which corresponds to the signal of interest (SOI). To improve the adaptability of the Capon beamformer, many robust adaptive beamformers have been proposed [7,8,9,10,11,12,13,14,15,16]. Vorobyov [7] developed a robust adaptive beamformer which is adapted to steering vector mismatch of arbitrary unknown signals. Li [8,9] used diagonal loading to optimize MVDR beamformers, which has excellent performance for the SOI power estimation. Kim [10] considered robust beamforming by using convex optimization to solve the worst-case signal-to-interference-plus-noise-ratio maximization problem. Beck [11] studied the generalized doubly constrained robust (GDCR) capon beamformer in which the uncertainty set is an ellipsoid. Aubry [12,13] designed a robust filter based on polynomial-time solution technique for radar pulse-doppler processing. Samuel [14] produced a wideband robust beamformer for passive sonar combined with the steered covariance matrix (STCM) method. 

Many DOA estimation techniques use narrowband signals for DOA estimation. Because the noise signals radiated by submarines and ships are typically wideband signals, it is necessary to extend these narrowband signal processing methods to wideband signal processing. At present, the most influential methods are the incoherent signal subspace (ISS) processing framework [19,20] and the coherent signal subspace (CSS) processing framework [21,22,23,24,25,26]. Zhang [19] presented an extension of test of orthogonality of projected subspaces (ETOPS) algorithm for wideband DOA estimation. Ma [20] proposed a speech signal classified energy-peak incoherent signal subspace method (SSC-EPISM) based on time-frequency sparseness of speech signal. In 1985, Wang [21] proposed CSS processing for DOA estimation. Hung [22,23] developed a class of robust focusing matrices for the CSS method. Li [24], Chen [25] and Bucris [26] studied robust methods based on CSS-MVDR for wideband signal DOA estimation. These methods can provide DOA estimation of wideband radiated ship noise. In the future, the environment of sea battlefield will become more complex. The radiated noise of underwater targets, such as submarines and surface ships, becomes weaker, but the noise of marine environments increases yearly. Because of their low azimuthal resolution and narrow detection range, the traditional DOA estimation methods, such as conventional beamformers and ISS-MVDR and CSS-MVDR beamformers, may not detect the target in time or provide accurate underwater target location information for the weapon system. In particular, researchers must solve the problem of improving DOA estimation performance for weak targets.

Starting in 2002, Cetin et al. [27,28] introduced sparsity theory into DOA estimation. By establishing a discrete sparse reconstruction model of the spatial angle, high-resolution DOA estimation is achieved by solving related optimization problems. In 2006, Candes et al. [29,30,31,32] proposed the theory of compressed sensing (CS) to establish a signal processing system which breaks the Nyquist sampling theorem. The DOA estimation methods based on CS show superior properties. Recently, the DOA estimation methods based on CS have been improved [33,34,35,36,37]. Kang et al. [36,37] proposed the ISS-CS method, which uses CS for DOA estimation and waveform restoration of underwater wideband targets. This method achieves better target resolution and detection performance than conventional methods. The above methods provide DOA estimation for narrow- or wideband targets based on the ISS processing framework.

For the DOA estimation problem of underwater wideband weak targets, we propose a novel method of DOA estimation based on coherent signal subspace processing and compressed sensing theory (CSS-CS). Using the processing framework of the CSS, the wideband frequency focusing is accomplished by a two-sided correlation transformation (TCT). Then, the wideband weak target DOA estimation is achieved by using the spatial sparsity of the target in the compressed sensing reconstruction algorithm. Finally, compared with the ISS-MVDR, CSS-MVDR and ISS-CS methods, the feasibility and effectiveness of the proposed CSS-CS method are verified by processing simulation data and marine trial data.

This paper is organized as follows: the receiving data model for the uniform linear array (ULA) is introduced in Section 2. The CSS-CS wideband target DOA estimation model, construction of focusing matrix, and compressed sensing DOA estimation are developed in Section 3. The results of simulation and marine trial data processing are presented in Section 4. Conclusions are provided in Section 5. 

## 2. Receiving Data Model for ULA

Following Cetin and Zhu [27,28,33], consider the far-field underwater acoustic environment with a ULA in which wideband signals from *N* sources are received by an *M* element hydrophone with spacing *d*. It is assumed that: (1) the target sources are wideband signals; (2) the ULA is located in the far field of the target source, and there is no mutual coupling between the elements; (3) the number of target sources is less than the number of array elements.

According to the frequency-domain data model of the ULA [36,37], the narrowband signal in the frequency domain with a center frequency of $f_{0}$ can be represented as:

(1) $$X(f_{0})=A(f_{0},\theta )S(f_{0})+N(f_{0})$$

where $X(f_{0})={\left[x_{1}(f_{0}),\cdots x_{m}(f_{0}),\cdots x_{M}(f_{0})\right]}^{T}$ $\in C^{M\times 1}$ is the frequency-domain representation of the received signal of the ULA, $x_{m}(f_{0})(m=1,\;2,\;\cdots ,\;M)$ denotes the signal received by the element *m* of the ULA, *M* is the number of hydrophone elements of the ULA, $f_{0}$ is the center frequency of the received signal, ${\left[\;\right]}^{T}$ is the matrix transpose operation, $S(f_{0})={\left[s_{1}(f_{0}),\cdots s_{n}(f_{0}),\cdots s_{N}(f_{0})\right]}^{T}$ $\in C^{N\times 1}$ is the frequency-domain representation of underwater acoustic signals, $s_{n}(f_{0})(n=1,\;2,\;\cdots ,\;N)$ denotes the underwater acoustic signal *n*, *N* is the number of underwater acoustic signals, $N(f_{0})={\left[n_{1}(f_{0}),\cdots n_{m}(f_{0}),\cdots n_{M}(f_{0})\right]}^{T}$ $\in C^{M\times 1}$ is the frequency-domain representation of additive noise, and $n_{m}(f_{0})(m=1,2,\cdots ,M)$ is the additive noise from the element *m* of the ULA. $A(f_{0},\theta )=\left[a(f_{0},\theta_{1}),\cdots a(f_{0},\theta_{n}),\cdots a(f_{0},\theta_{N})\right]$ $\in C^{M\times N}$ is the array manifold of the ULA, where $a(f_{0},\theta_{n})$ $\in C^{M\times 1}$ is the response vector (or direction vector) of the ULA to the signal *n* with center frequency $f_{0}$, and $\theta_{n}$ denotes the direction of the signal *n*. The response vector $a(f_{0},\theta_{n})$ is expressed as:

(2) $$a(f_{0},\theta_{n})={\left[e^{-j2\pi \;f_{0}\tau_{n1}},\cdots e^{-j2\pi \;f_{0}\tau_{nm}},\cdots e^{-j2\pi \;f_{0}\tau_{nM}}\right]}^{T}\;n\;=\;1,\;2,\;\cdots ,\;N$$

where $\tau_{nm}$ denotes the relative delay of the received signal *n* to the element *m* of the ULA. If the array element 1 is the reference element, then $\tau_{nm}$ is:

(3) $$\tau_{nm}=\frac{(m-1)d\sin\theta_{n}}{c}=\frac{(m-1)d\sin\theta_{n}}{\lambda \text{​}f_{0}}$$

where *c* is the underwater acoustic velocity, *λ* is the wavelength of the acoustic wave, and *d* is the spacing of the ULA elements. Putting Equation (3) into Equation (2), we can obtain the array manifold:

(4) $$\begin{array}{ll} A(f_{0},\theta ) & =\left[a(f_{0},\theta_{1}),\;\cdots a(f_{0},\theta_{n}),\cdots \;a(f_{0},\theta_{N})\right]\\ & =\left[\begin{array}{ccccc} 1 & \cdots & 1 & \cdots & 1\\ e^{-j\frac{2\pi }{\lambda }d\sin\theta_{1}} & \cdots & e^{-j\frac{2\pi }{\lambda }d\sin\theta_{n}} & \cdots & e^{-j\frac{2\pi }{\lambda }d\sin\theta_{N}}\\ \vdots & \vdots & \vdots & \vdots & \vdots \\ e^{-j\frac{2\pi }{\lambda }(M-1)d\sin\theta_{1}} & \cdots & e^{-j\frac{2\pi }{\lambda }(M-1)d\sin\theta_{n}} & \cdots & e^{-j\frac{2\pi }{\lambda }(M-1)d\sin\theta_{N}} \end{array}\right] \end{array}$$

From the expression (4) for the array manifold $A(f_{0},\theta )$, the elements of the array manifold correspond to individual pairs of target direction and receiver.

## 3. CSS-CS Wideband Target DOA Estimation

### 3.1. Wideband Target DOA Estimation Model

**Figure 1:**
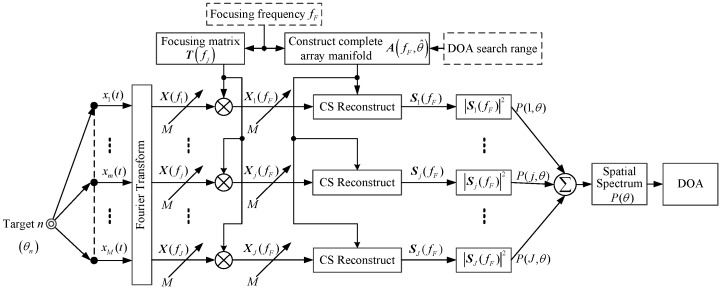
Wideband target DOA estimation model.

The core idea of the CSS method proposed by Wang and Kaveh [21] is focused transformation. The signal subspace of each frequency component of the broadband signal is focused to the signal subspace of the same frequency, and then the DOA estimation is carried out via narrowband signal processing. Using CSS processing, an underwater wideband target DOA estimation model is shown in Figure 1. First, a Fourier transform is used to convert time domain signals into frequency domain signals with *J* sub-bands. Second we designe a bank of filters to transform the frequency domain signals with different frequency signal subspaces to *J* signals with signal subspaces on the same focused frequency $f_{F}$. In this step, the filters use focusing matrix $T\left(f_{j}\right)$ which is closely related to each sub-band signal and the focused frequency $f_{F}$. The matrix $T\left(f_{j}\right)$, which is very important for subsequent operations and directly affects the estimation performance of DOA, has to be properly constructed. Third, the complete array manifold $A_{a}(f_{F},\hat{\theta })$ is constructed under the focused frequency $f_{F}$, and the compressed sensing reconstruction algorithm is used to solve the spatial spectrum P(j,θ) of *J* signals. Finally, the total spatial spectrum P(θ) is estimated by summation of P(j,θ), and the target DOA estimation is achieved by searching for the peak value of the total spatial spectrum P(θ).

We suppose that $x_{m}(t)\;(m=1,\;2,\;\cdots ,\;M)$ is the time-domain signal received by the element *m* of the ULA. By using the Fourier transform, $x_{m}(t)\;$ can be converted into multiple frequency-domain sub-band array signals $X(f_{j})\;={\left[X_{1}(f_{j}),\cdots X_{m}(f_{j}),\cdots X_{M}(f_{j})\right]}^{T}\;$ $\in C^{M\times 1}$ (j = 1, 2, ⋯, J), where *J* is the number of sub-bands, $f_{j}$ denotes the center frequency of the *j* sub-band, and $X_{m}(f_{j})\left(m\;=\;1,\;2,\;\cdots ,\;M\right)$ denotes the *j* sub-band signal of the element *m*, where *M* is the number of element hydrophones of the ULA. $X(f_{j})$ is the matrix form of the *j* sub-band signals and the ULA received data model can be expressed as:

(5) $$X(f_{j})=A(f_{j},\theta )S(f_{j})+N(f_{j})$$

where $S(f_{j})={\left[S_{1}(f_{j}),\cdots S_{n}(f_{j}),\cdots S_{N}(f_{j})\right]}^{T}$ $\in C^{N\times 1}$ denotes the *j* sub-band of the underwater acoustic signals, $S_{n}(f_{j})$ is *j* sub-band of the signal *n*, $N(f_{j})={\left[N_{1}(f_{j}),\cdots N_{m}(f_{j}),\cdots N_{M}(f_{j})\right]}^{T}$ $\in C^{M\times 1}$ denotes the *j* sub-band of the additive noise, $N_{m}(f_{j})$ is the *j* sub-band of the additive noise by the element *m* of the ULA, and $A(f_{j},\theta )=[a(f_{j},\theta_{1}),\cdots a(f_{j},\theta_{n}),\cdots ,a(f_{j},\theta_{N})]$ $\in C^{M\times N}$ is the *j* sub-band of the ULA array manifold, where $a(f_{j},\theta_{n})$ $\in C^{M\times 1}$ denotes the *j* sub-band response vector (or direction vector) of the ULA to the signal *n*.

In (5), the array manifold $A\left(f_{j},\theta \right)$ is related to the sub-band frequency $f_{j}$. If we can transform different $A\left(f_{j},\theta \right)$ into $A\left(f_{F},\theta \right)$ that are only dependent on the frequency $f_{F}$, such as Equation (6), via a focusing matrix $T\left(f_{j}\right)$ that transforms the array manifold at each frequency point to the same frequency, then the narrowband methods can be used to estimate DOA. That is:

(6) $$A\left(f_{F},\theta \right)=T\left(f_{j}\right)A\left(f_{j},\theta \right)$$

where, $T\left(f_{j}\right)$ $\in C^{M\times M}$ is the focusing matrix, and $f_{F}$ is the focused frequency.

We use the focusing matrix transform to change the different-frequency signal subspaces into the same frequency signal subspace:

(7) $$X_{j}\left(f_{F}\right)=T\left(f_{j}\right)X\left(f_{j}\right)\;$$

where, $T\left(f_{j}\right)$ $\in C^{M\times M}$ denotes the focusing matrix for *j* sub-band signal, $X\left(f_{j}\right)\;$ $\in C^{M\times 1}$ is the *j* sub-band signal, $X_{j}\left(f_{F}\right)$ $\in C^{M\times 1}$ denotes the *j* sub-band focused signal with the same frequency signal subspace, and $f_{F}$ is the focused frequency. From Equation (7), the received signal X(f)  with signal subspaces of different frequencies are mapped to the $X_{j}\left(f_{F}\right)$ with signal subspaces on the same reference frequency $f_{F}$.

The spatial spectrum of each sub-band can be obtained by using the narrow band beamforming method for $X_{j}\left(f_{F}\right)$, and the total spatial spectrum can be obtained by summing the spatial spectrum of each sub-band. The target DOA can be obtained by searching for the peak value of the total spatial spectrum. In this way, the CSS-MVDR method uses the MVDR narrowband beamformer and CSS-CS method uses the CS narrowband beamformer after the focusing transformation.

### 3.2. Construction of Focusing Matrix

The performance of the DOA estimation is directly affected by the construction of the focusing matrix, so the selection of the focusing criterion and the solution of the focusing matrix are key issues for the CSS method. The Two-sided Correlation Transformation (TCT) [38,39,40] algorithm selects the focusing matrix by using the relationship between the noiseless data of each frequency point. The covariance matrix is calculated on both sides of equations of type (7), the following formula can be obtained:

(8) $$C_{X}\left(f_{F},\theta \right)=T\left(f_{j}\right)C_{X}\left(f_{j},\theta \right)T^{H}\left(f_{j}\right)$$

where ${\left[\;\right]}^{H}$ is the matrix conjugate transpose operation, $C_{X}\left(f_{j},\theta \right)$ $\in C^{M\times M}$ denotes the covariance matrix of $X\left(f_{j},\theta \right)$, $C_{X}\left(f_{j},\theta \right)=X\left(f_{j},\theta \right)X^{H}\left(f_{j},\theta \right)$, and $C_{X}\left(f_{F},\theta \right)$ $\in C^{M\times M}$ denotes the covariance matrix of $X\left(f_{F},\theta \right)$, with $C_{X}\left(f_{F},\theta \right)=X\left(f_{F},\theta \right)X^{H}\left(f_{F},\theta \right)$.

Considering the error and avoiding focusing loss, the problem of solving the focusing matrix $T\left(f_{j}\right)$ can be transformed into a constrained minimization problem [38,39]:

(9) $$\begin{array}{l} \underset{T\left(f_{j}\right)}{\min}{\left\|C_{X}\left(f_{F}\;,\theta \right)-T\left(f_{j}\right)C_{X}\left(f_{j},\theta \right)T^{H}\left(f_{j}\right)\right\|}_{F}\\ \;s.t.\;T\left(f_{j}\right)T^{H}\left(f_{j}\right)=I\;(j\;=\;1,\;2,\;\cdots ,\;J) \end{array}$$

where ${\left\|\;\right\|}_{F}$ is the Frobenius norm operation. From [38], the error of Equation (9) is given by:

(10) $$\begin{array}{ll} \varepsilon & ={\left\|C_{X}\left(f_{F}\;,\theta \right)-T\left(f_{j}\right)C_{X}\left(f_{j},\theta \right)T^{H}\left(f_{j}\right)\right\|}_{F}\\ & ={\left\|C_{X}\left(f_{F}\;,\theta \right)\right\|}_{F}+{\left\|C_{X}\left(f_{j},\theta \right)\right\|}_{F}-2ℜ\;\mathrm{tr}\left(C_{X}\left(f_{F}\;,\theta \right)T\left(f_{j}\right)C_{X}^{H}\left(f_{j},\theta \right)T^{H}\left(f_{j}\right)\right) \end{array}$$

where tr(·) denotes the trace of a matrix, and ℜ denotes the real part of a complex number. Minimization of Equation (10) with respect to the choice of $T\left(f_{j}\right)$ is identical to maximization of:

(11) $$\underset{T\left(f_{j}\right)}{\max}\;ℜ\;\mathrm{tr}\left(C_{X}\left(f_{F}\;,\theta \right)T\left(f_{j}\right)C_{X}^{H}\left(f_{j},\theta \right)T^{H}\left(f_{j}\right)\right)$$

From the lemma in [38], the best norm solution of Equations (9) and (12) is the focusing matrix:

(12) $$T\left(f_{j}\right)=U\left(f_{F}\right)U^{H}\left(f_{j}\right)$$

where $U\left(f_{F}\right)$ $\in C^{M\times 1}$ is the principal eigenvector matrix of $C_{X}\left(f_{F},\theta \right)$, and $U\left(f_{j}\right)$ $\in C^{M\times 1}$ is the principal eigenvector matrix of $C_{X}\left(f_{j},\theta \right)$.

### 3.3. Compressed Sensing DOA Estimation

Compressed sensing (CS) theory has been widely studied in the field of DOA estimation [27,28,29,30,31,32,33,34,35,36,37], and good estimation performance has been achieved. Based on the compressed sensing and array receiving data model, the basic method of narrowband target DOA estimation is as follows.

We divide the whole space into $\left\{{\hat{\theta }}_{1},\cdots {\hat{\theta }}_{h},\cdots {\hat{\theta }}_{H}\}(H>>N\right)$, with intervals sufficiently small for the desired DOA resolution, and assume that every possible direction ${\hat{\theta }}_{h}(h\;=\;1,\;2,\;\cdots ,\;H)$ corresponds to a potential target signal ${\hat{s}}_{h}$. Thus, *H* target signals $S_{a}(f_{0})\;=\;{\left[{\hat{s}}_{1}(f_{0}),\cdots {\hat{s}}_{h}(f_{0}),\cdots {\hat{s}}_{H}(f_{0})\right]}^{T}$ $\in C^{H\times 1}$ are constructed, and the array manifolds can be represented as [18,19,20,21,22,23,24,25,26,27]:

(13) $$\begin{array}{l} A_{a}(f_{0},\hat{\theta })=\left[a(f_{0},{\hat{\theta }}_{1}),\;\cdots a(f_{0},{\hat{\theta }}_{h}),\cdots a(f_{0},{\hat{\theta }}_{H})\right]\\ ={\left[\begin{array}{ccccc} 1 & \cdots & 1 & \cdots & 1\\ e^{-j\frac{2\pi }{\lambda }d\sin{\hat{\theta }}_{1}} & \cdots & e^{-j\frac{2\pi }{\lambda }d\sin{\hat{\theta }}_{h}} & \cdots & e^{-j\frac{2\pi }{\lambda }d\sin{\hat{\theta }}_{H}}\\ \vdots & \vdots & \vdots & \vdots & \vdots \\ e^{-j\frac{2\pi }{\lambda }(M-1)d\sin{\hat{\theta }}_{1}} & \cdots & e^{-j\frac{2\pi }{\lambda }(M-1)d\sin{\hat{\theta }}_{h}} & \cdots & e^{-j\frac{2\pi }{\lambda }(M-1)d\sin{\hat{\theta }}_{H}} \end{array}\right]}_{M\times H} \end{array}$$

where $A_{a}(f_{0},\hat{\theta })$ $\in C^{M\times H}$ denotes the complete array manifold, and the received-narrowband-signals model for ULA can be represented as follow:

(14) $$X_{a}(f_{0})=A_{a}(f_{0},\hat{\theta })S_{a}(f_{0})+N_{a}(f_{0})$$

where $S_{a}(f_{0})$ $\in C^{H\times 1}$ is a sparse representation of the signal space, and $N_{a}(f_{0})$ $\in C^{H\times 1}$ is a sparse representation of the noise space. The array manifold $A(f_{0},\theta )$ to be estimated is a subset of the complete array manifold $A_{a}(f_{0},\hat{\theta })$. The power of $S_{a}(f_{0})$ should be such that only the direction of the corresponding target is strong, while the other directions should display a smaller value. In the compressed sensing model [30], $X_{a}(f_{0})$ can be seen as an observation sequence, $A_{a}(f_{0},\hat{\theta })$ as a perception matrix, $S_{a}(f_{0})$ as a sparse coefficient component for solving, and $N_{a}(f_{0})$ as measurement noise. Therefore, the following convex optimization problem can be used to solve the signal space estimation [29,30,31,32,33]:

(15) $$\begin{array}{l} \min{\left\|{\hat{S}}_{a}(f_{0})\right\|}_{1}^{}\;\\ s.t.\;X_{a}(f_{0})=A_{a}(f_{0},\hat{\theta }){\hat{S}}_{a}(f_{0})+N_{a}(f_{0}) \end{array}$$

where ${\left\|\;\right\|}_{1}$ is the 1 norm operation, and ${\hat{S}}_{a}(f_{0})$ denotes the eatimated signal in the frequency domain, $N_{a}(f_{0})$ denotes additive noise in the frequency domain. 

From (7) and (13), a convex optimization model for the estimated sub-band target signal ${\hat{S}}_{j}(f_{F})$ using the focused sub-band signal $X_{j}\left(f_{F}\right)$ can be obtained:

(16) $$\begin{array}{l} \min{\left\|{\hat{S}}_{j}(f_{F})\right\|}_{1}^{}\;\\ s.t.\;X_{j}(f_{F})=A_{a}(f_{F},\hat{\theta }){\hat{S}}_{j}(f_{F})+N_{j}(f_{F}) \end{array}$$

where ${\hat{S}}_{j}(f_{F})$ denotes the *j* focused sub-band target signal, $X_{j}\left(f_{F}\right)$ denotes the *j* focused sub-band received signal and $N_{j}(f_{F})$ denotes the *j* focused sub-band additive noise. From ${\hat{S}}_{j}(f_{F})$, the corresponding signal power of each DOA can be calculated, and the corresponding spatial spectrum estimation of the *j* sub-band can be obtained, namely:

(17) $$P(j,\theta )={\left|{\hat{S}}_{j}(f_{F})\right|}^{2}$$

where P(j,θ) denotes the corresponding spatial spectrum estimation of the *j* sub-band signal. Summing over all sub-bands P(j,θ), the total spatial spectral estimation is obtained:

(18) $$P(\theta )=\sum_{j=1}^{J}P(j,\theta )$$

where P(θ) denotes the spatial spectral estimation.

The DOA of the target can be estimated by searching for the peak value of the spatial spectrum P(θ).

In summary, the specific steps of CSS-CS are as follows:

Step 1. The Fourier transform of $X\left(t\right)={\left[x_{1}\left(t\right),\cdots x_{m}\left(t\right),\cdots ,x_{M}\left(t\right)\right]}^{T}$ is used to convert time domain data into frequency domain data $X(f_{j})\;={\left[X_{1}(f_{j}),\cdots X_{m}(f_{j}),\cdots X_{M}(f_{j})\right]}^{T}\;$(j= 1, 2, ⋯, J).
Step 2. Choose the focus frequency $f_{F}$ and solve the focusing matrix $T\left(f_{j}\right)$ from Equation (10).
Step 3. From Equation (7), the $X(f_{j})\;$ with signal subspaces of different frequencies are mapped to the $X_{j}\left(f_{F}\right)$ with signal subspaces on the same reference frequency $f_{F}$.
Step 4. Divide the whole space into $\left\{{\hat{\theta }}_{1},\cdots {\hat{\theta }}_{h},\cdots {\hat{\theta }}_{H}\}(H>>N\right)$ with the desired direction interval and construct the complete array manifold $A_{a}(f_{F},\hat{\theta })$ from Equation (13).
Step 5. From Equation (16), estimate the sub-band target signal ${\hat{S}}_{j}(f_{F})$ from the focused sub-band received signal $X_{j}\left(f_{F}\right)$.
Step 6. From Equation (17), calculate the spatial spectrum P(j,θ) of each *j* sub-band.
Step 7. From Equation (18), estimate the total spatial spectrum P(θ) by summing the spatial spectra of all sub-bands.
Step 8. Estimate the DOA of the target by searching for the peak of P(θ).

## 4. Data Verification and Analysis

### 4.1. Simulation Data Analysis

Some simulations are conducted in this section to evaluate the performance of the proposed algorithms. We consider a 32-element ULA with element spacing *d* = 1 m. The sampling frequency is $f_{s}$ = 25 kHz, the number of snapshots is *K* = 25,000, and the scan range is (−90°, 90°) with a 0.1° step. In addition, two actual wideband ship-radiated noise signals are taken as the original target signals with DOA 5° and 10°, and identical powers. We assume that the received signals are polluted by additive white Gaussian noise with *SNR =* 3 dB and the acoustic velocity is 1500 m/s.

#### 4.1.1. Analysis of the Focused Frequency Selection Problem

**Figure 2:**
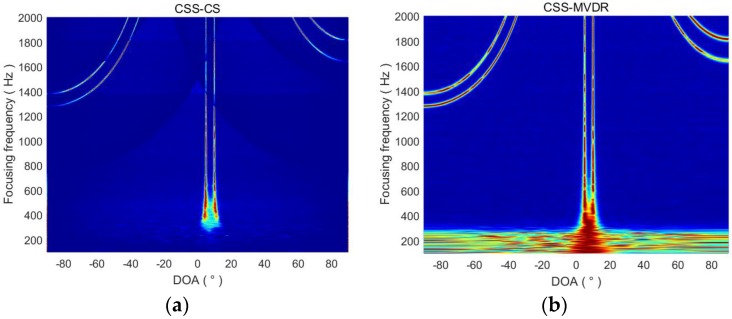
DOA estimation results of CSS-CS and CSS-MVDR in terms of focused frequencies (targets at 5° and 10°). (**a**) DOA estimation results of CSS-CS; (**b**) DOA estimation results of CSS-MVDR.

**Figure 3:**
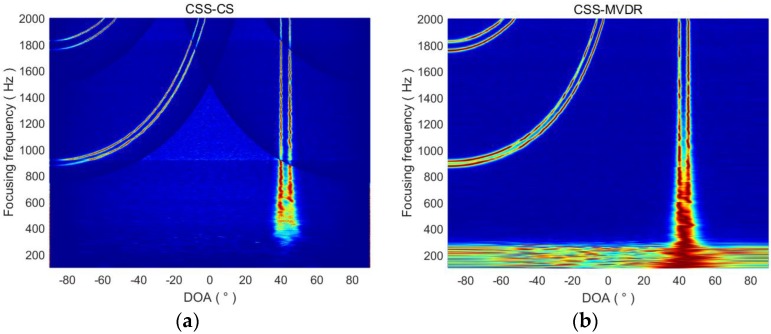
DOA estimation results of CSS-CS and CSS-MVDR in terms of focused frequencies (targets at 40° and 45°). (**a**) DOA estimation results of CSS-CS; (**b**) DOA estimation results of CSS-MVDR.

From Section 3.2, the focused frequency selection is very important for the CSS-CS method. The focused frequency selection problem is studied in this section. We let the focused frequency range from 100 Hz to 2000 Hz. When two targets are located at 5° and 10°, or 40° and 45°, the DOA estimation results of CSS-CS and CSS-MVDR are shown in Figure 2 and Figure 3, respectively. In Figure 2, the axis represents the DOA estimation of the target signal, and the horizontal axis indicates the change of the focused frequency. In Figure 2a, when the focused frequency is lower than 300, the CSS-CS method cannot complete estimation of the two target signals. With the increased focused frequency, the CSS-CS method gradually becomes able to distinguish the two signals. With the increasing of the focused frequency, the CSS-CS method can identify two signals gradually, and the DOA estimation curve also becomes finer. It indicates that the azimuth resolution of the CSS-CS method increases with the increased focused frequency. However, when the focused frequency is greater than 1200 Hz, we find that 4 targets were estimated to be located at 5°, 10°, −60° and −70°. Those at 5° and 10° are the real targets, while −60° and −70° are the false targets. When the focused frequency is greater than 1800 Hz, we find that 6 targets were estimated to be located at −50°, −40°, 5°, 10°, 60° and 80°. Those at 5° and 10° are the real targets, and others are false targets. In Figure 2b, a similar phenomenon can also be found for the CSS-MVDR method. In Figure 3, this phenomenon is more obvious as the azimuth of the two real target signals increases. Thus, we can come to the conclusion that, with the increased focused frequency, the directional resolution and performance of the two methods will gradually improve, but when the focused frequency is higher than the half wavelength frequency $f_{Half}$ of the ULA, an ambiguity in the DOA estimation will appear:

(19) $$f_{Half}=c/\lambda_{Half}=c/2d$$

where $f_{Half}$ is related to the spacing of ULA elements.

In this section, the element spacing *d* = 1 m, so $f_{Half}$ = 750 Hz. When the targets are located at 5° and 10°, and the focused frequency is higher than 1200 Hz, the DOA estimation will become blurred. When the targets are located at 40° and 45°, and the focused frequency is higher than 800 Hz, the DOA estimation will become blurred. Thus, with the increase of the target azimuth angle, the focused frequency must be set lower so as to avoid the DOA ambiguity estimate. However, because in practice the target angle is unknown, a focused frequency must be chosen that works for all angles. According to the simulation results, the focused frequency setting should be inferred from the element spacing, i.e., $f_{F}\;=\;f_{Half}=\;c/2d$, which can not only ensure the algorithm has the highest directional resolution, but also overcome the problem of DOA ambiguity. Therefore, in the subsequent simulation experiments, the focused frequency is selected as 750 Hz, the analysis frequency range of the broadband signal is set from 400 Hz to 800 Hz, the sub-band bandwidth is 40 Hz, and the sub-band interval is 10 Hz.

#### 4.1.2. Performance Analysis of Directional Resolution

**Figure 4:**
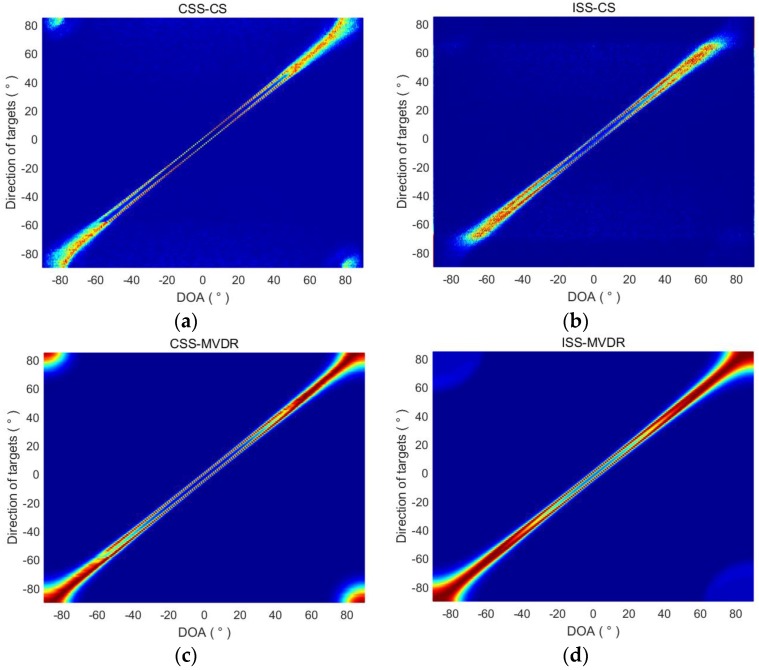
DOA estimation results of CSS-CS, ISS-CS, CSS-MVDR and ISS-MVDR in terms of target directions. (**a**) DOA estimation results of CSS-CS; (**b**) DOA estimation results of ISS-CS; (**c**) DOA estimation results of CSS-MVDR; (**d**) DOA estimation results of ISS-MVDR

In this section, the influence of target direction on the performance of the algorithm is studied. We assume that the two targets are spaced 5 degrees apart, varying from −90° and −85° to 85° and 90°, respectively. The DOA estimation results of CSS-CS, ISS-CS, CSS-MVDR and ISS-MVDR are shown in Figure 4.

Figure 4 shows that the DOA estimation curves of CSS-CS and CSS-MVDR for two targets are very fine, indicating that the two methods have higher directional resolution than the ISS ones. It also shows that the CSS-CS and CSS-MVDR methods have a wider range of directional detection, and the DOA estimation can be achieved when the two targets are in the range of −60° to 60°. For the ISS-CS and ISS-MVDR methods, the DOA estimation of the two targets can only be carried out in the range of −50° to 50°.

**Figure 5:**
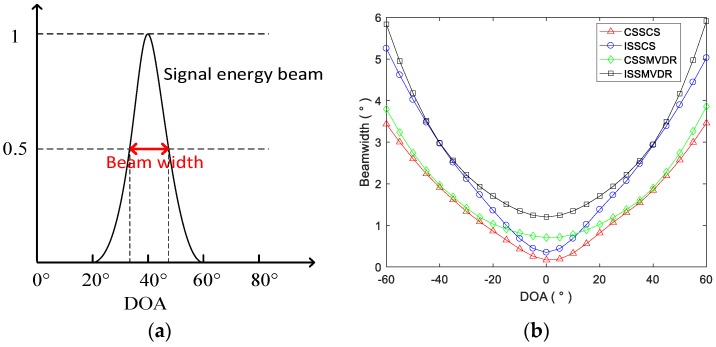
The definition of beam width and the four methods’ beam widths for DOA estimation. (**a**) The definition of beam width; (**b**) The beam widths of the four methods for DOA estimation.

The definition of beam width is shown in Figure 5a. It shows that the beam width is the angle range measured between the half-maximums of the signal energy beam in the spatial spectrum, when the DOA is estimated. The beam width shows the resolution of two targets in different directions in space. The beam widths of the four methods for DOA estimation of wideband targets in the range of −60° to 60° are shown in Figure 5b. It shows that, when the target is in the range of −20° to 20°, the directional resolution of CSS-CS is higher than the other three methods, ISS-CS and CSS-MVDR are intermediate, and ISS-MVDR is the worst. When the target is in the range of −60° to −20° and 20° to 60°, the directional resolution of the algorithms under the CSS processing framework is higher than the algorithms under the ISS processing framework; moreover, the resolution of the CSS-CS is slightly higher than that of the CSS-MVDR, and the resolution of the ISS-CS is slightly higher than that of the ISS-MVDR.

#### 4.1.3. Performance Analysis of DOA Estimation for Weak Targets

**Figure 6:**
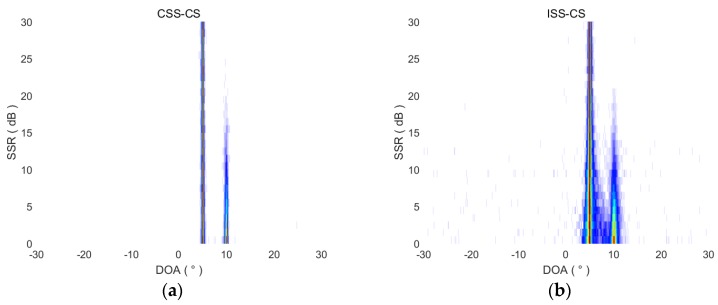

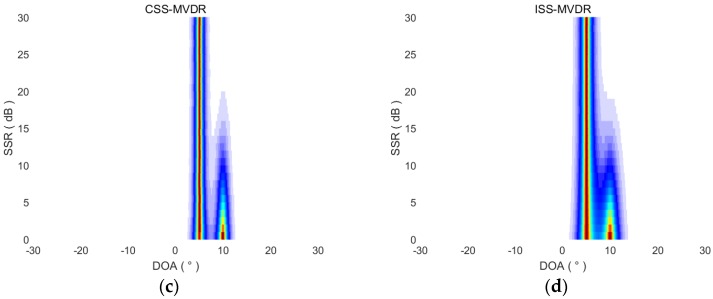
DOA estimation results of CSS-CS, ISS-CS, CSS-MVDR and ISS-MVDR in terms of SSRs. (**a**) DOA estimation results of CSS-CS in terms of SSRs; (**b**) DOA estimation results of ISS-CS in terms of SSRs; (**c**) DOA estimation results of CSS-MVDR in terms of SSRs; (**d**) DOA estimation results of ISS-MVDR in terms of SSRs.

For the DOA estimation of weak targets, this section focuses on the impact of signal to signal ratio (SSR) on the performance of the algorithms. In the previous sections, the simulation is carried out with identical strengths for the two target signals, but in reality, the power of several targets will not be identical. The value of SSR is defined as follows:

(20) $$SSR=10\log_{10}\left(P_{s1}/P_{s2}\right)$$

where $P_{s1}$ and $P_{s2}$ are the two signal powers, and the unit of SSR is dB. We let the 5° signal power remain unchanged and the 10° signal power gradually weaken. When the SSR of the 5° signal and the 10° signal changes from 0 dB to 30 dB, the DOA estimation of the four methods is as shown in Figure 6. It shows that, when the SSR is higher than 20 dB, CSS-CS can also complete the detection and DOA estimation for the weak 10° signal. The performance of CSS-CS is better than the other three methods, ISS-CS and CSS-MVDR are intermediate, and ISS-MVDR is the worst.

When a Monte Carlo simulation is used to analyze the performance of the algorithm, the root mean square error (RMSE) is generally used to evaluate the performance of the DOA estimation:

(21) $$RMSE_{\theta_{n}}=\sqrt{\frac{1}{K}\sum_{k=1}^{K}{\left(\theta_{n}-{\hat{\theta }}_{nk}\right)}^{2}}\;n=1,2,\cdots ,N$$

where K is the number of Monte Carlo simulations, $\theta_{n}$ is the DOA of the target *n*, and ${\hat{\theta }}_{nk}$ is the DOA estimation of the target *n* estimated in the *k* simulation.

**Figure 7:**
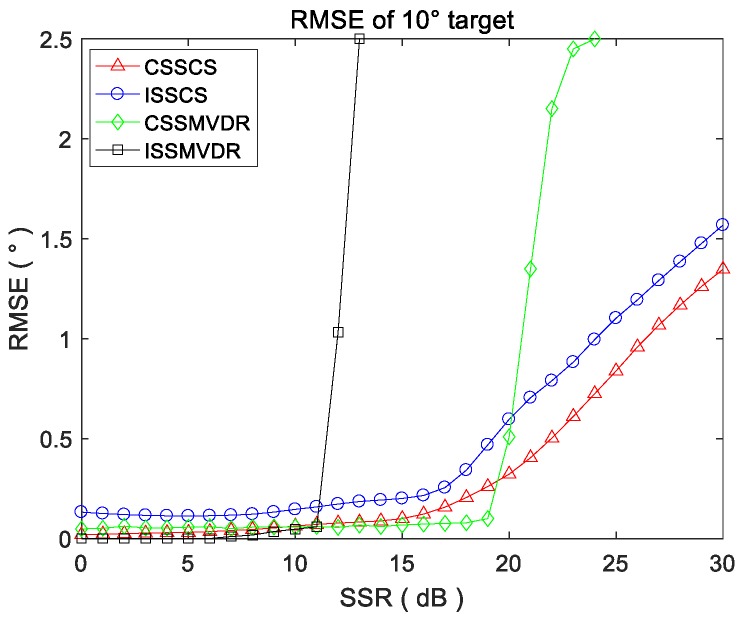
NMSE curves of four methods for DOA estimation of 10° target in terms of SSRs.

Since the 5° target power remains unchanged, the four methods can effectively estimate its DOA, but the power of the 10° target decreases gradually, so it is significant to estimate the RMSE of four methods for the 10° target’s DOA. After 100 Monte-Carlo simulation experiments, the RMSE curves of four methods for DOA estimation of the 10° target under different SSR conditions are drawn in Figure 7. Since the direction interval between the two targets is only 5°, the estimation is invalid when the DOA estimation error is greater than 2.5°. Thus, when SSR changes from 0 dB to 30 dB, the RMSE curves are drawn within 2.5° in Figure 7.

This figure shows that the detection and DOA estimation ability of CSS-CS for weak targets is better than the other three methods; when the SSR is less than 25 dB, the DOA estimation error of the CSS-CS is less than 1°. The weak target detection ability of ISS-CS is second, CSS-MVDR is third, and ISS-MVDR is the worst because it can only estimate the weak target with SSR less than 12 dB.

#### 4.1.4. Performance Analysis of the Snapshot Number

**Figure 8:**
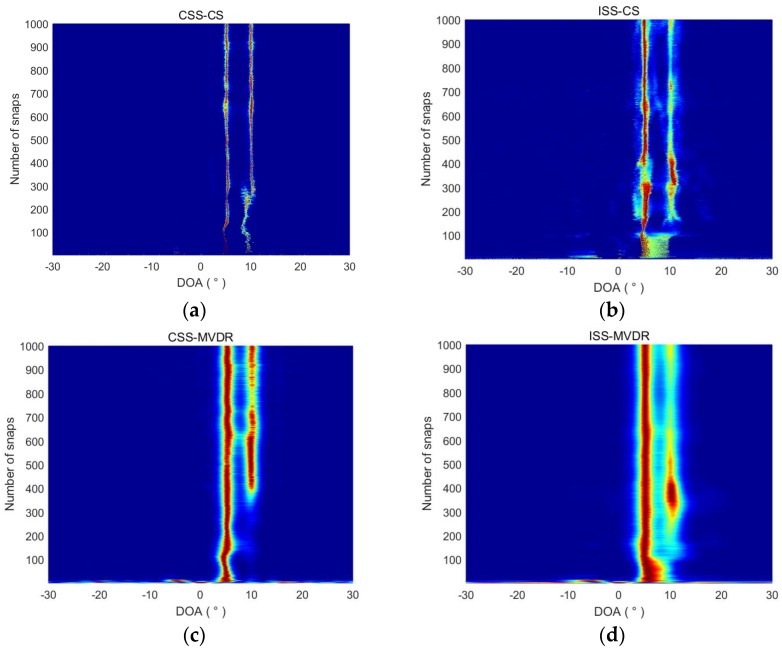
DOA estimation of the four methods under different snapshot numbers. (**a**) DOA estimation of CSS-CS under different snapshot numbers; (**b**) DOA estimation of ISS-CS under different snapshot numbers; (**c**) DOA estimation of CSS-MVDR under different snapshot numbers; (**d**) DOA estimation of ISS-MVDR under different snapshot numbers.

The influence of the snapshot number on the performance of the algorithms is studied in this section. We suppose that the snapshot number varies from 2 to 1000. The DOA estimation of the four methods is shown in Figure 8. It can be seen that, in order to estimate the two targets’ DOA effectively, the CSS-CS method needs fewer snapshots than the other three methods. When the snapshot number is greater than 300, CSS-CS can accurately estimate the DOA of the two targets, but when the snapshot number is less than 300, CSS-CS not only can accurately estimate the DOA of the 5° target, but also can estimate the DOA of the 10° target, albeit with a certain error. In order to complete the two targets’ DOA estimation, the least snapshot number for ISS-CS, CSS-MVDR, and ISS-MVDR is 100, 200, and 300 respectively.

#### 4.1.5. Towed Line Array Sonar Simulation Data Analysis

This section uses the actual towed line array sonar to perform the simulation experiment, mainly considering the influence of the towboat’s own radiation noise and the marine environment. We assume that a 32-element ULA with element spacing *d* = 1 m is towed by a tugboat, the jamming intensity of the tugboat is 10 dB, the environmental noise level is about 0 dB (relative value) and there are three target signals in different directions. The direction of target 1 is −60° with intensity −18 dB, the direction of target 2 is −25° with intensity −25 dB, and the direction of target 3 is −22° with intensity −20 dB. The sampling frequency is *fs* = 25 kHz, the data acquisition time is 13 s.

**Figure 9:**
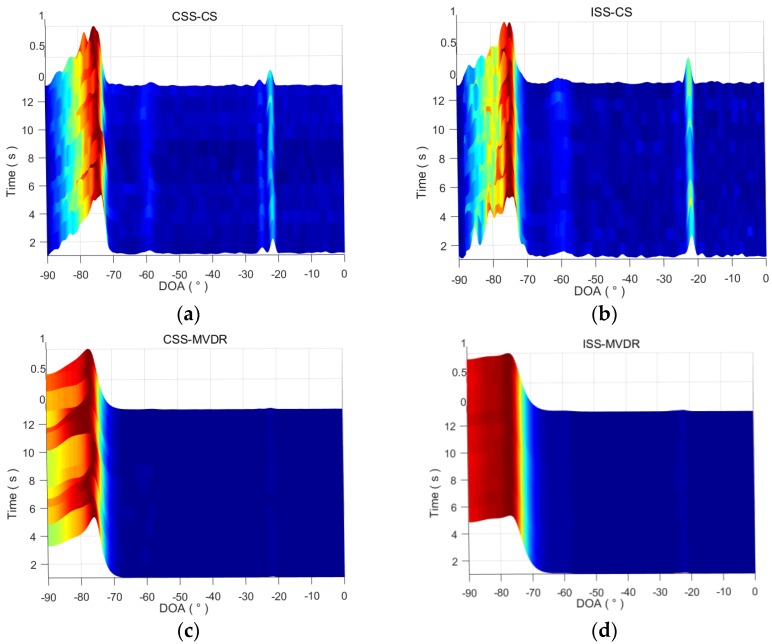
Three-dimensional DOA time tracks estimated by four methods. (**a**) Three-dimensional DOA time tracks estimated by CSS-CS; (**b**) Three-dimensional DOA time tracks estimated by ISS-CS; (**c**) Three-dimensional DOA time tracks estimated by CSS-MVDR; (**d**) Three-dimensional DOA time tracks estimated by ISS-MVDR.

The three-dimensional DOA time tracks estimated by CSS-CS, ISS-CS, CSS-MVDR and ISS-MVDR are drawn in Figure 9. From Figure 9a, CSS-CS can estimate the DOA time tracks of the three targets, and the DOA time tracks of the −22° target is clearer than that of the −25° and −60° targets. From Figure 9b, ISS-CS can estimate the DOA time tracks of the −22° and −60° targets, but the DOA time tracks of the −25° target is unclear. From Figure 9c,d, the DOA time tracks of the three targets is blurred, and CSS-MVDR and ISS-MVDR cannot detect the three targets clearly. It also shows that, whether using ISS or CSS, the target detection capability of CS is better than MVDR; namely, the ISS-CS method is better than the ISS-MVDR, and the CSS-CS method is better than the CSS-MVDR.

**Figure 10:**
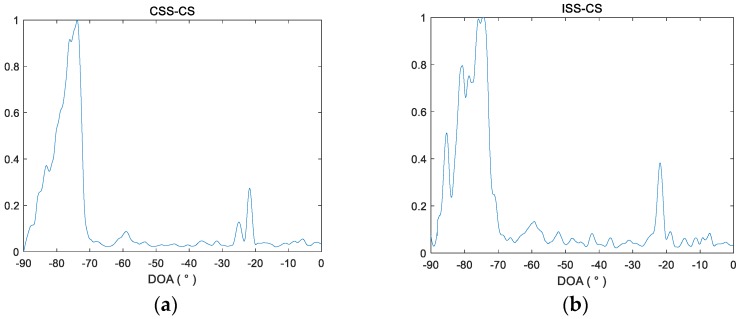

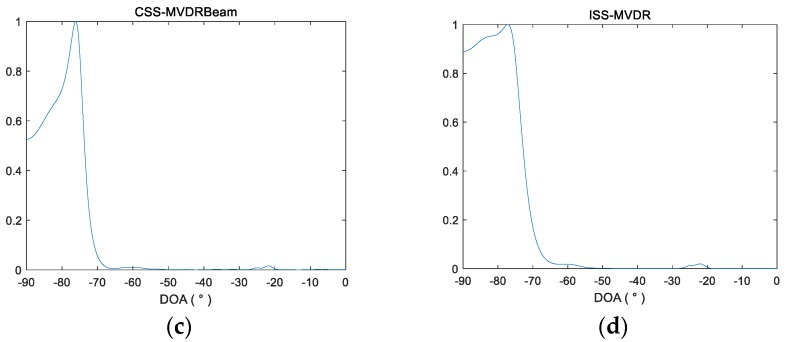
Spatial beam spectra after processing the first second data by four methods. (**a**) Spatial beam spectra after processing the first second data by CSS-CS; (**b**) Spatial beam spectra after processing the first second data by ISS-CS; (**c**) Spatial beam spectra after processing the first second data by CSS-MVDR; (**d**) Spatial beam spectra after processing the first second data by ISS-MVDR.

The spatial spectra after processing the first second data by four methods are drawn in Figure 10. It shows that the CSS-MVDR and ISS-MVDR methods can only perceive the targets at −60° and −23°, and the estimated signal power in the corresponding direction of the spatial spectrum is very weak. ISS-CS can detect the two weak targets at −60° and −23°. CSS-CS can detect the three weak targets in −60°, −25° and −23°, and the spatial spectrum of the three targets obtained by CSS-CS is clearer, with better detection performance for the −25° target. Therefore, the overall performance of CSS-CS is better than the other three methods.

### 4.2. Marine Trial Data Verification and Analysis

**Figure 11:**
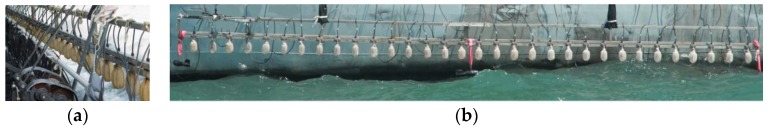
The ULA used in the marine trial. (**a**) Partial enlargement of the ULA; (**b**) The photograph of the ULA before it was put into water.

We carried out a marine trial near the Laopian Island off the Tiger Beach in Bohai Bay, Dalian, China. The depth of the sea area is 40–50 m, and the ocean current velocity is about 3 knots. The array used in the marine trial is a rigid horizontal ULA with 32 elements, as shown in Figure 11. During the marine trial, the 1st, 2nd, and 32nd element were damaged, so the number of effective array elements was 29. The hydrophone spacing is 0.225 m and the receiving array depth is about 9 m.

**Figure 12:**
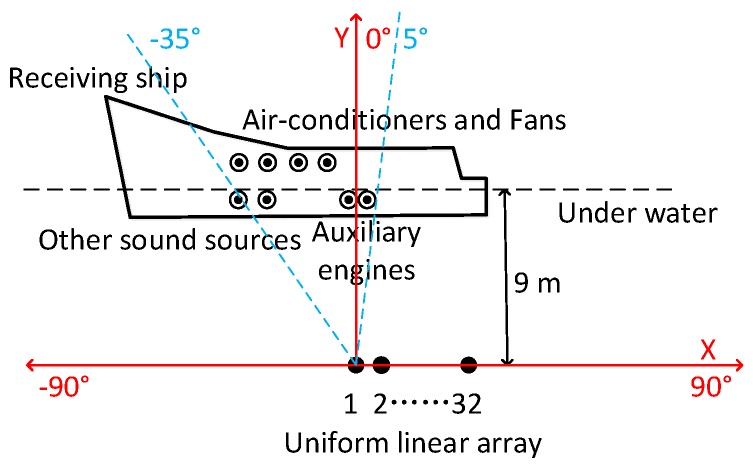
The general distribution of self-radiated noise from the receiving ship.

The receiving array was put down from the side of the receiving ship, not the towed one. During the marine trial, the receiving ship was anchored, but the auxiliary engine always generated electricity, mutiple air conditioners, fans and other equipments were working too, thus the platform produced very strong self-radiated noise from distributed sources. The general distribution of self-radiated noise from the receiving ship is shown in Figure 12. 

**Figure 13:**
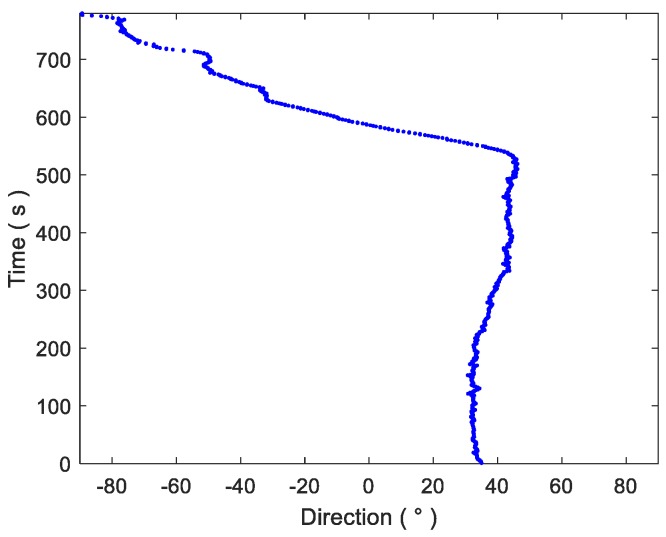
GPS direction information of target ship.

**Figure 14:**
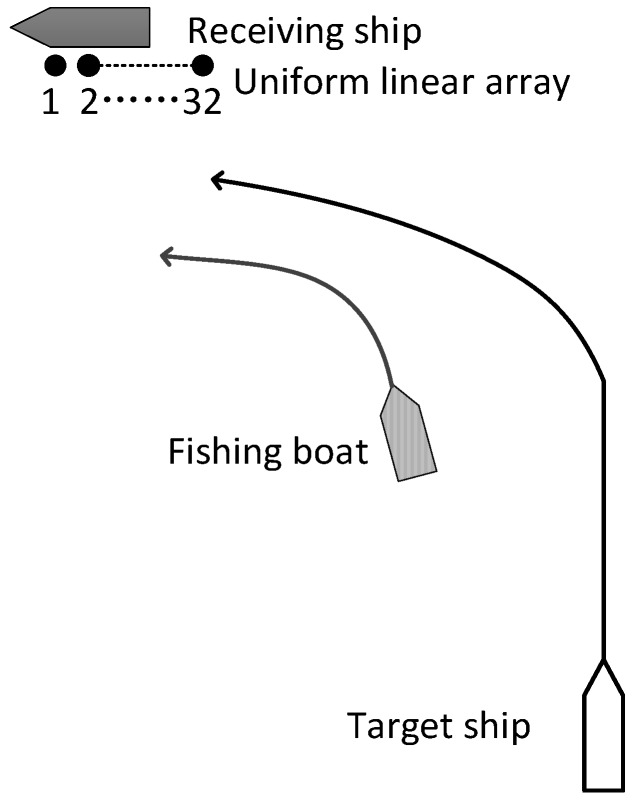
Schematic diagram of the maritime situation during marine trial.

The target ship was the same type as the receiving ship, with 1.5 m draft, speed 16 knots. The target ship approached the receiving ship from about 7.4 km away, and then passed close by the receiving vessel. The direction of the target ship as determined by GPS is shown in Figure 13. At the same time, as the target ship moved, there were also fishing boats and other targets within visual range. The schematic diagram of the maritime situation during the marine trial is shown in Figure 14. In the first few seconds before the trial, the target ship was in the low-speed steering phase, and there was a fishing boat about 3 km away from the receiving ship. Therefore, in the first few seconds of the trial, the target ship signal was weaker than the fishing boat signal and in almost the same direction.

**Figure 15:**
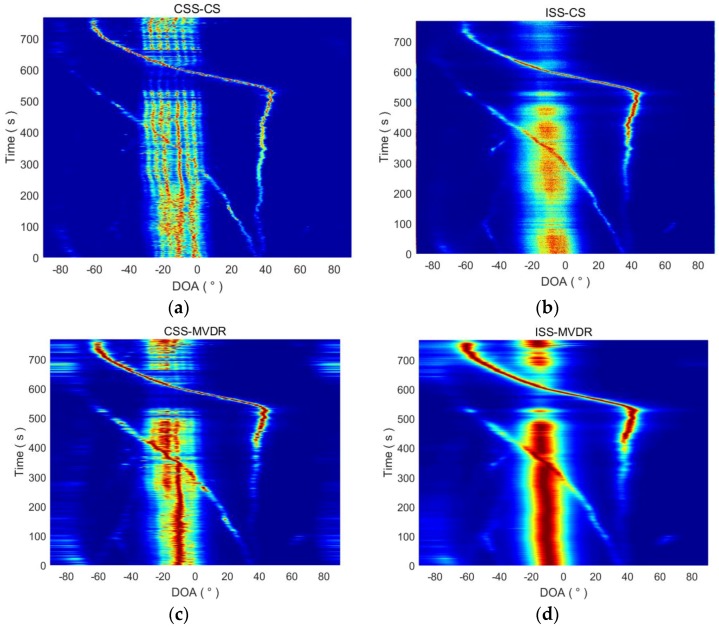
The entire DOA estimation time tracks of the four methods. (**a**) The entire DOA estimation time tracks of CSS-CS; (**b**) The entire DOA estimation time tracks of ISS-CS; (**c**) The entire DOA estimation time tracks of CSS-MVDR; (**d**) The entire DOA estimation time tracks of ISS-MVDR.

The sampling frequency of the system is 48 kHz. According to the simulation results from Section 4. A, which suggest a focused frequency of $f_{F}=c/2d$, since the hydrophone spacing is 0.225 m, the focused frequency for CSS-CS and CSS-MVDR is set at 3333 Hz in subsequent data processing. In addition, the directional scan range is (−90°, 90°) with a 0.0625° step, the analysis frequency range of broadband signal is set from 500 Hz to 3333 Hz, the sub-band bandwidth is 40 Hz, and the sub-band interval is 10 Hz. The final DOA estimation results of the four methods are shown in Figure 15. It shows that the overall target movement trend, which is also called the DOA time track, can be effectively estimated by the four methods. We found that the noise radiated from the receiving ship is mainly distributed within the range of −35° to 5°, which is consistent with the situation described in Figure 12. Figure 15 also shows that there are multiple target DOA time trackes, which look like stripes appearing in the azimuth area of the receiving ship in the course estimated by the CSS-CS and CSS-MVDR methods. That occurs because the radiated noise of the receiving ship is not a single sound source, but is composed of multiple noise signals produced by the auxiliary generators, air conditioners, fans and other instruments that were working continuously. These devices were relatively close to the receiving array, and for the high resolution method, the radiated noise of the receiving ship cannot be seen as a point source. The CSS-CS and CSS-MVDR methods have high azimuth resolution and can achieve DOA estimation of the different noise sources in the receiving ship, so there are multiple target DOA time tracks, which look like stripes, appearing in the CSS-CS and CSS-MVDR results. However, the target azimuth resolution of the ISS-CS and ISS-MVDR methods is lower, so that the DOA estimation of different radiating noise sources on the receiving ship cannot be completed, and thus there are no stripes in the ISS-CS and ISS-MVDR results. This also verifies that the CSS-CS method has a higher azimuth resolution than the other three methods.

**Figure 16:**
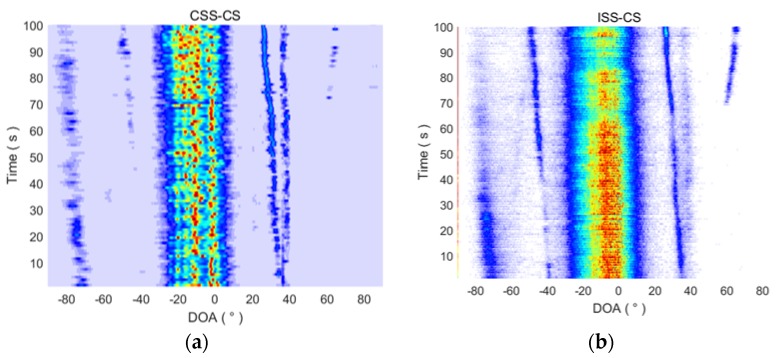

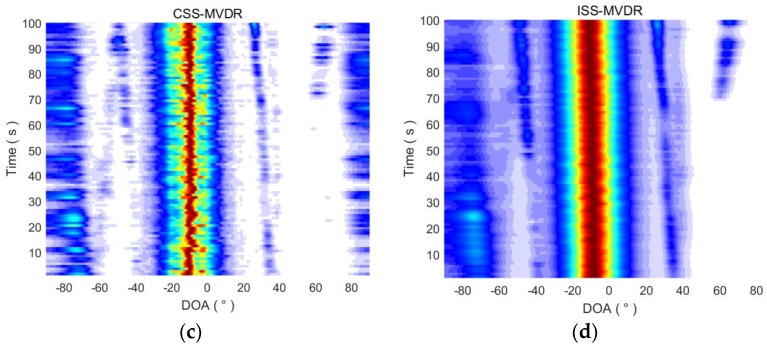
The DOA estimation time tracks of the four methods in the first 100 s. (**a**) The DOA estimation time tracks of CSS-CS in the first 100 s; (**b**) The DOA estimation time tracks of ISS-CS in the first 100 s; (**c**) The DOA estimation time tracks of CSS-MVDR in the first 100 s; (**d**) The DOA estimation time tracks of ISS-MVDR in the first 100 s.

The DOA results from 1 to 100 s are magnified in Figure 16, with a modified color distribution. In the first 100 s, the target ship is far away from the receiving ship and the energy is weak, so it is a typical weak target in the sea. The CSS-CS method can estimate the DOA of the target ship more accurately, and its performance is also better than the other three methods.

## 5. Conclusions

Based on the coherent signal subspace processing framework for wideband signals and compressed sensing theory for DOA estimation, a CSS-CS method is proposed for underwater wideband weak-target DOA estimation. The effectiveness of the proposed method in DOA estimation is verified by processing simulation data and marine trial data. CSS-CS can improve the DOA resolution and enhance the weak target detection ability of a towed line array sonar. Compared with the traditional method, this method has higher direction resolution, wider DOA estimation range, fewer required snapshots and more accurate detection ability for weak targets. However, due to the simplicity of the test equipment and the conditions, the DOA estimation accuracy and resolution of the proposed method in the actual marine background need further testing and verification. In the future, CSS-CS can be applied to the improvement of towed line array sonar arrays, underwater target detection and identification.
